# Long term results of postoperative Intensity-Modulated Radiation Therapy (IMRT) in the treatment of Squamous Cell Carcinoma (SCC) located in the oropharynx or oral cavity

**DOI:** 10.1186/s13014-015-0561-y

**Published:** 2015-12-04

**Authors:** M. Hoffmann, L. Saleh-Ebrahimi, F. Zwicker, P. Haering, A. Schwahofer, J. Debus, P.E. Huber, F. Roeder

**Affiliations:** Clinical Cooperation Unit Molecular Radiation Oncology, German Cancer Research Center (DKFZ), Heidelberg, Germany; Department of Radiation Oncology, University Hospital of Munich (LMU), Marchioninistr. 15, 81377 Munich, Germany; Department of Radiation Physics, German Cancer Research Center (DKFZ), Heidelberg, Germany; Department of Radiation Oncology, University of Heidelberg, Heidelberg, Germany; Clinical Cooperation Unit Radiation Oncology, German Cancer Research Center (DKFZ), Heidelberg, Germany

**Keywords:** Oropharyngeal cancer, Postoperative, IMRT

## Abstract

**Background:**

To report our long-term results with postoperative intensity-modulated radiation therapy (IMRT) in patients suffering from squamous-cell carcinoma (SCC) of the oral cavity or oropharynx.

**Methods:**

Seventy five patients were retrospectively analyzed. Median age was 58 years and 84 % were male. 76 % of the primaries were located in the oropharynx. Surgery resulted in negative margins (R0) in 64 % of the patients while 36 % suffered from positive margins (R1). Postoperative stages were as follows: stage1:4 %, stage2:9 %, stage3:17 %, stage4a:69 % with positive nodes in 84 %. Perineural invasion (Pn+) and extracapsular extension (ECE) were present in 7 % and 29 %, respectively. All patients received IMRT using the ste*p*-and-shoot approach with a simultaneously integrated boost (SIB) in 84 %. Concurrent systemic therapy was applied to 53 patients, mainly cisplatin weekly.

**Results:**

Median follow-u*p* was 55 months (5–150). 13 patients showed locoregional failures (4 isolated local, 4 isolated neck, 5 combined) transferring into 5-year-LRC rates of 85 %. Number of positive lymph nodes (*n* > 2) and presence of ECE were significantly associated with decreased LRC in univariate analysis, but only the number of nodes remained significant in multivariate analysis. Overall treatment failures occurred in 20 patients (9 locoregional only, 7 distant only, 4 combined), transferring into 3-and 5-year-FFTF rates of 77 % and 75 %, respectively. The 3-and 5-year-OS rates were 80 % and 72 %, respectively. High clinical stage, high N stage, number of positive nodes (*n* > 2), ECE and Pn1 were significantly associated with worse FFTF and OS in univariate analysis, but only number of nodes remained significant for FFTF in multivariate analysis. Maximum acute toxicity was grade 3 in 64 % and grade 4 in 1 %, mainly hematological or mucositis/dysphagia. Maximum late toxicity was grade 3 in 23 % of the patients, mainly long-term tube feeding dependency.

**Conclusion:**

Postoperative IMRT achieved excellent LRC and good OS with acceptable acute and low late toxicity rates. The number of positive nodes (*n* > 2) was a strong prognostic factor for all endpoints in univariate and the only significant factor for LRC and FFTF in multivariate analysis. Patients with feeding tubes due to postoperative complications had an increased risk for long-term feeding tube dependency.

## Background

The main treatment modalities for head and neck cancer are surgery and radiation therapy [1–5]. While very early stages can be successfully treated with one of those modalities alone, surgical candidates with more advanced stages usually require the combination of both [5] to achieve satisfactory locoregional control. Further on, enhancement of radiation therapy by simultaneous application of chemotherapy has been shown to be advantageous according to randomized trials in the presence of several risk factors especially positive or close resection margins and nodal involvement with extracapsular extension (ECE) [3, 4, 6]. However, in most of these trials patients with different tumor locations have been included and treated uniformly, although tumor localization might influence treatment opportunities and outcome both regarding oncological and functional endpoints [7, 8]. For example, surgical opportunities in oral cavity and oropharyngeal tumors can be limited with regard to radicality because of unacceptable functional deficits [9], which fueled even the investigation of neoadjuvant chemoradiation approaches specifically in those cases [10]. In fact several authors reported different outcomes for oral cavity or oropharyngeal tumors compared to other head and neck sites [8, 11]. However, both surgical and radiation therapy techniques have evolved in the last decades. While the surgical approach has been improved for example by the widened use of reconstructions with microvascular free flaps [9], the introduction of intensity-modulated radiation therapy (IMRT) represents a major progress in radiation therapy. As shown by various dosimetric and clinical studies, IMRT allows improved target coverage and reduced dose to organs at risk at the same time which consequently leads to less acute and late toxicities [12, 13]. Further on, IMRT offers a simple solution to apply slightly accelerated doses in areas of high risk [14–16] like the surgical cavity without increasing the number of fractions (so-called simultaneously integrated boost concept, SIB). Taken together, little long-term data exists specifically addressing the subgrou*p* of patients with oral cavity or oropharyngeal primaries treated with postoperative IMRT with or without chemotherapy depending on the presence of risk factors established in randomized trials using conventional techniques. We therefore retrospectively evaluated our patients treated in this setting with regard to outcome, toxicity and possible prognostic factors to gain a more specific insight into the long-term clinical behavior of this patient subgrou*p* in the era of modern radiation techniques.

## Methods

### Patient characteristics

We retrospectively analyzed our patients with squamous cell cancer of the oral cavity or the oropharynx who have been treated with postoperative intensity-modulated radiation therapy after gross complete resection at our institution between 2000 and 2010. Oral cavity cancer was defined as primary tumor located in the mucosal surface of lip, floor of mouth, oral tongue, buccal mucosa, lower and upper gingival, hard palate and retromolar trigone, according to UICC^6th^ definition. Oropharyngeal cancer was defined as primary tumor located in the soft palate, tonsil, base of tongue and lateral or posterior wall of the pharynx between soft palate and hyoid according to UICC^6th^ definition. Patients with distant spread or locally recurrent disease at presentation, gross residual disease after resection, prior radiation therapy of the head and neck region, induction chemotherapy or non-squamous cell cancer histology were excluded. The remaining 75 patients formed the basis of the current analysis. Median age was 58 years (35–85) and 84 % were male. 61 % of the primaries were located in the oropharynx. Surgery resulted in microscopically negative margins (R0) in 64 % of the patients while 36 % suffered from positive margins (R1). All patients received ipsilateral (45 %) or bilateral neck dissections (55 %). Postoperative tumor stages (UICC^6th^ 2002) were distributed as follows: stage 1: 3 %, stage 2:7 %, stage 3: 13 %, stage 4a: 52 % with positive nodes in 84 % of the patients. Grading was G1 in 3 %, G2 in 57 % and G3 in 40 %. Perineural invasion (Pn+) was present in 7 %, extracapsular extension (ECE) in 29 %. For detailed patient characteristic see Table 1.

**Table 1:** Patient and Treatment characteristics

	n	%		n	%
Age			Follow-up		
median	58 yrs		median	55 mo	
min	35 yrs		min	5 mo	
max	85 yrs		max	150 mo	
Gender			RT break > 3d		
male	63	84	yes	3	4
female	12	16	no	72	96
Localisation			SIB		
oral cavity	18	24	yes	63	84
oropharynx	57	76	no	12	16
pT stage			Number of beams		
pT1	23	31	median	9	
pT2	36	48	min	5	
pT3	9	12	max	10	
pT4a	7	9			
			TD nodal		
pN stage			median	54 Gy	
pN0	12	16	min	50 Gy	
pN1	12	16	max	60 Gy	
pN2a	5	7			
pN2b	39	52	TD Boost		
pN2c	7	9	median	66 Gy	
			min	60 Gy	
clinical stage (UICC6)			max	70.4 Gy	
stage 1	3	4			
stage 2	7	9	SD nodes		
stage 3	13	17	median	1.8 Gy	
stage 4a	52	69	min	1.8 Gy	
			max	2 Gy	
Grading					
G1	2	3	SD Boost		
G2	43	57	median	2.2 Gy	
G3	30	40	min	2 Gy	
			max	2.33 Gy	
ECE					
yes	22	29	Chemotherapy		
no	53	71	yes	53	71
			no	22	29
Perineural invasion					
Pn0	70	93	CHT scheme/compl.		
Pn1	5	7	Cis weekly	47	89^a^
			Carbo/5-FU	5	9^a^
Number of pos. nodes			Cetuximab	1	1^a^
* n* ≤ 2	44	59	>80 % of scheduled	41	77^a^
* n* > 2	31	41	<80 % of scheduled	12	23^a^
Resection margin			Neck dissection		
R0	48	64	ipsilateral	34	45
R1	27	36	bilateral	41	55

*Yrs* years, *min* minimum, *max* maximum, *n* number, %:percentage, *UICC*6 union international contre le cancer staging manual 6th edition, *ECE* extracapsular extension, *pos*. positive, *RT* radiation therapy, *d* days, *mo* months, *TD* total dose, *SD*: single dose, *CHT* chemotherapy, compl.:completion, *Cis* cisplatin, *Carbo* carboplatin, 5-*FU*5-fluorouracil, *SIB* simultaneously integrated boost, ^a^percentage of 53 patients with CHT

### Work-u*p* and surgery

Initial work-u*p* prior to surgery included clinical and laboratory examination, computed tomography (CT) and/or magnetic resonance imaging (MRI) of the head and neck, endoscopy with histological confirmation, chest x-ray or CT and abdominal ultrasound or CT. Surgery included various techniques for gross primary tumor removal with fla*p* reconstructions if technically needed and ipsi-or bilateral neck dissection according to the principles of head and neck cancer surgery. Indication for postoperative radiation was seen in locally advanced primary tumors (T3/4), positive lymph nodes (N+) or incomplete resection. In case of incomplete resection or positive lymph nodes with extracapsular extension, patients were scheduled for simultaneous platin-based chemotherapy if medically fit. Surgery attempted gross complete removal of the primary by various techniques and ipsi-or bilateral neck dissection. Radiation was planned to be initiated 4–8 weeks after surgery if primary wound closure was achieved.

### Radiation therapy

**Fig. 1:**
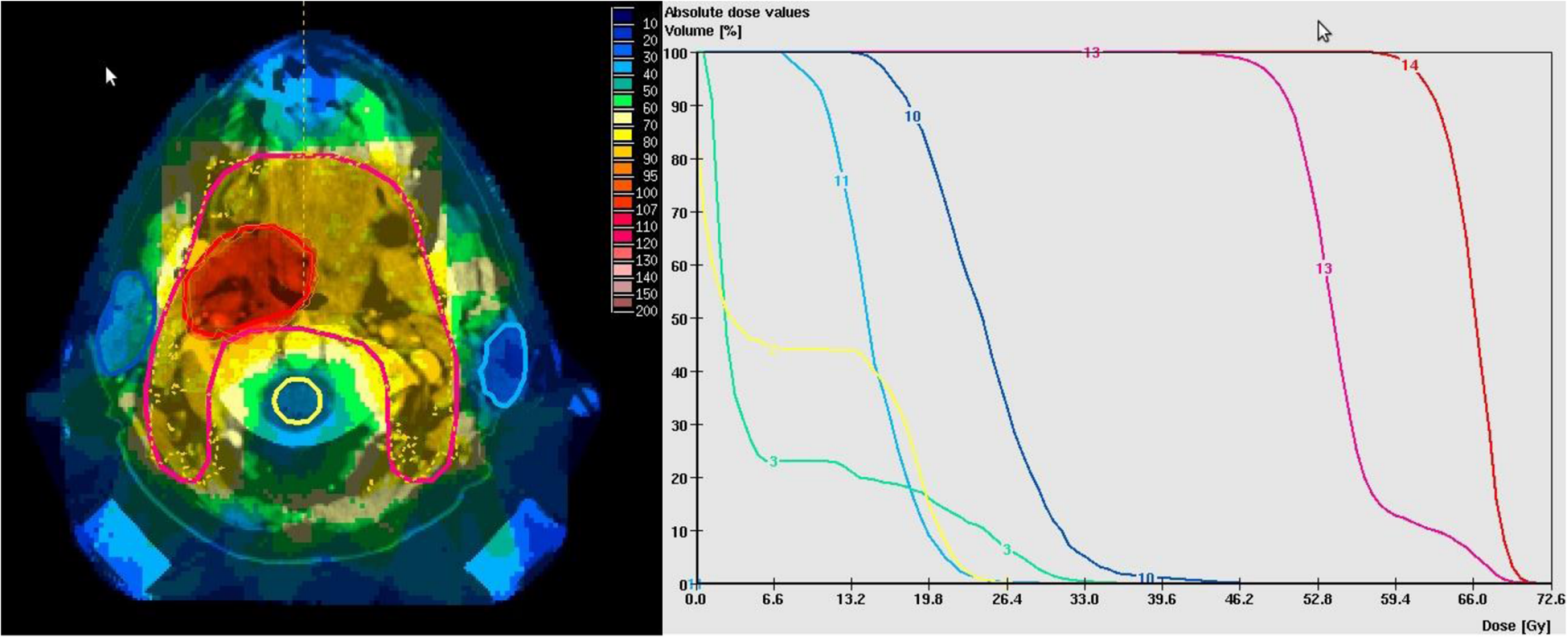
Example of dose distribution and DVH. Treatment plan for a patient with postoperative chemoradiation with cisplatin weekly, prescription dose 54 Gy to nodal region (single dose 1.8 Gy) and simultaneously integrated boost with 66 Gy (single dose 2.2 Gy), left: dose distribution, right: DVH, 2: myelon, 3: brainstem, 10/11: parotid glands, 13: nodal PTV, 14: boost PTV

All patients received postoperative IMRT using the ste*p* and shoot approach. The technique used in our institution has been described previously [14, 17, 18]. Briefly, all patients were fixed in an individually manufactured precision head mask made of Scotch cast (3 M, St. Paul, Minneapolis, MN) and a vacuum pillow for the body. With this immobilization system attached to the stereotactic base frame, contrast-enhanced CT-images were performed with a slice thickness of at least 3 mm. Target volume definition differed slightly over time but usually the primary clinical target volume included the surgical tumor bed with a safety margin of 1 cm and the bilateral regional lymph nodes areas (retro-, parapharyngeal, cervical nodes level Ib-V). Secondary CTVs (Boost) covered the surgical tumor bed and the regions of involved lymph nodes with extracapsular extension. A PTV margin of 3–5 mm was added manually to the CTVs. Margins could be reduced in case of directly adjacent organs at risk. Inverse treatment planning was performed using the KonRad and VIRTOUS software developed at the German Cancer Research center (DKFZ). EBRT was delivered by linear accelerators with 6 or 15 MV photons using an integrated motorized multileaf collimator (MLC) in ste*p*-and shoot technique. Since the introduction of a kV-CT on rails in 2002, all patients received image guidance (with the possibility for replanning if necessary) at least once a week. The total doses were prescribed to the median of the target volume and usually the 95 % isodose surrounded the PTV. A simultaneously integrated boost concept (SIB) was used in the majority of patients (84 %). According to this concept, the boost volume (surgical bed, nodal regions with ECE) should be covered with 66 Gy (single dose 2.2 Gy) while the nodal areas should receive 54 Gy (single dose 1.8 Gy) in 30 fractions. An example of a three dimensional dose distribution including a DVH sample is shown in Fig. 1. At least one parotid gland was spared (mean dose lower than 26 Gy). In patients with sequential boost concepts, conventional fractionation was used (1.8-2 Gy). Chemotherapy schedules varied slightly over time, but in general patients were scheduled for concurrent platin-based systemic therapy in case of microscopic residual disease (R1 resection), close margin resection or if extracapsular extension was present.

### Follow-up

Regular follow-u*p* visits took place either at our or at the referring center. At our institution, patients were scheduled for follow u*p* visits every 3 months for the first 2 years, every six months for the following 3 years and annually thereafter. Each visit included at least clinical examination and CT or MRI of the head and neck. In case of evidence for locoregional recurrence or distant spread, additional tests or imaging modalities were performed to confirm or exclude disease progression at the discretion of the treating physician. Missing data were completed by calling the patients or the treating physician.

### Definition of events

Local control (LC) was defined as absence of tumor regrowth in the region of the primary tumor. Neck control (NC) was defined as absence of tumor regrowth in the bilateral regional nodes. Locoregional control (LRC) was defined as absence of local or neck recurrence. In patients without further assessment of LC/NC/LRC, for example after development of distant spread, the date of the last information about the local/neck/regional status was used for calculation. Distant control (DC) was defined as absence of distant failure. Freedom from treatment failure (FFTF) was defined as absence of regional or distant failure. All patients were (re-)-staged according to UICC 6th edition. Postoperative complications, acute and late side effects were reported as documented in the patient charts. Acute toxicity was scored according to Common Toxicity Criteria version 3.0 (CTCAE V3.0) from the start of radiation therapy until 3 months of follow up. Late toxicity was scored according to CTCAE 3.0 thereafter until the end of follow-up. If multiple occurence was documented, the most severe grade of a specific event was used for grading. Disease related functional impairments present prior to the start of chemoradiation were scored as toxicity only if worsening occurred. Xerostomia was scored as subjectively assessed by the patients and graded according to Radiation Therapy Oncology Grou*p* (RTOG)/European Organization for Research and Treatment (EORTC) radiation morbidity scoring criteria [19].

### Statistical and ethical considerations

Time to event data was calculated from the first day of radiation treatment until the last follow u*p* information or until death using the Kaplan-Meier method. Subgroups were compared using the log-rank test. Parameters with *p* < 0.1 in univariate analysis were entered into a Cox regression model for multivariate analysis. Differences were considered statistically significant for a *p*-value of ≤ 0.05. The study is in compliance with the Declaration of Helsinki (Sixth Revision, 2008). Furthermore the study was approved by the Independent Ethics Committee of the Medical Faculty Heidelberg (Ref. Nr.: S-170/2012). All patients gave written informed consent before treatment initiation.

## Results

The median follow u*p* for the entire cohort was 55 months (5–150) and 62 months in survivors (12–150). Only two of the surviving patients had a follow-u*p* interval less than 2 years. Radiation Treatment breaks >3 days were needed in 3 patients (one due to skin/mucosal toxicity, one due to suspected fla*p* necrosis and one due to acute appendicitis). Median radiation treatment time was 43 days (36–57 days). 77 % of the patients scheduled for chemotherapy received at least 80 % of the planned chemotherapy dose.

### Locoregional control

**Fig. 2:**
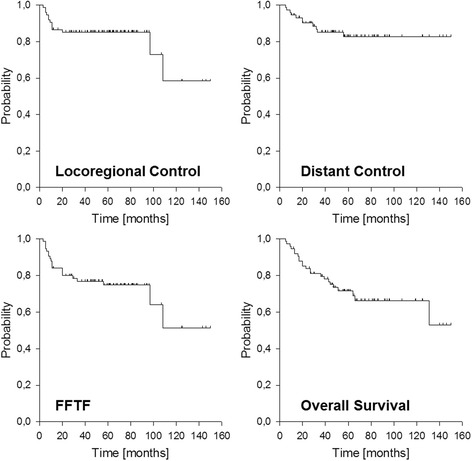
Outcome of the entire cohort. FFTF: freedom from treatment failure

**Fig. 3:**
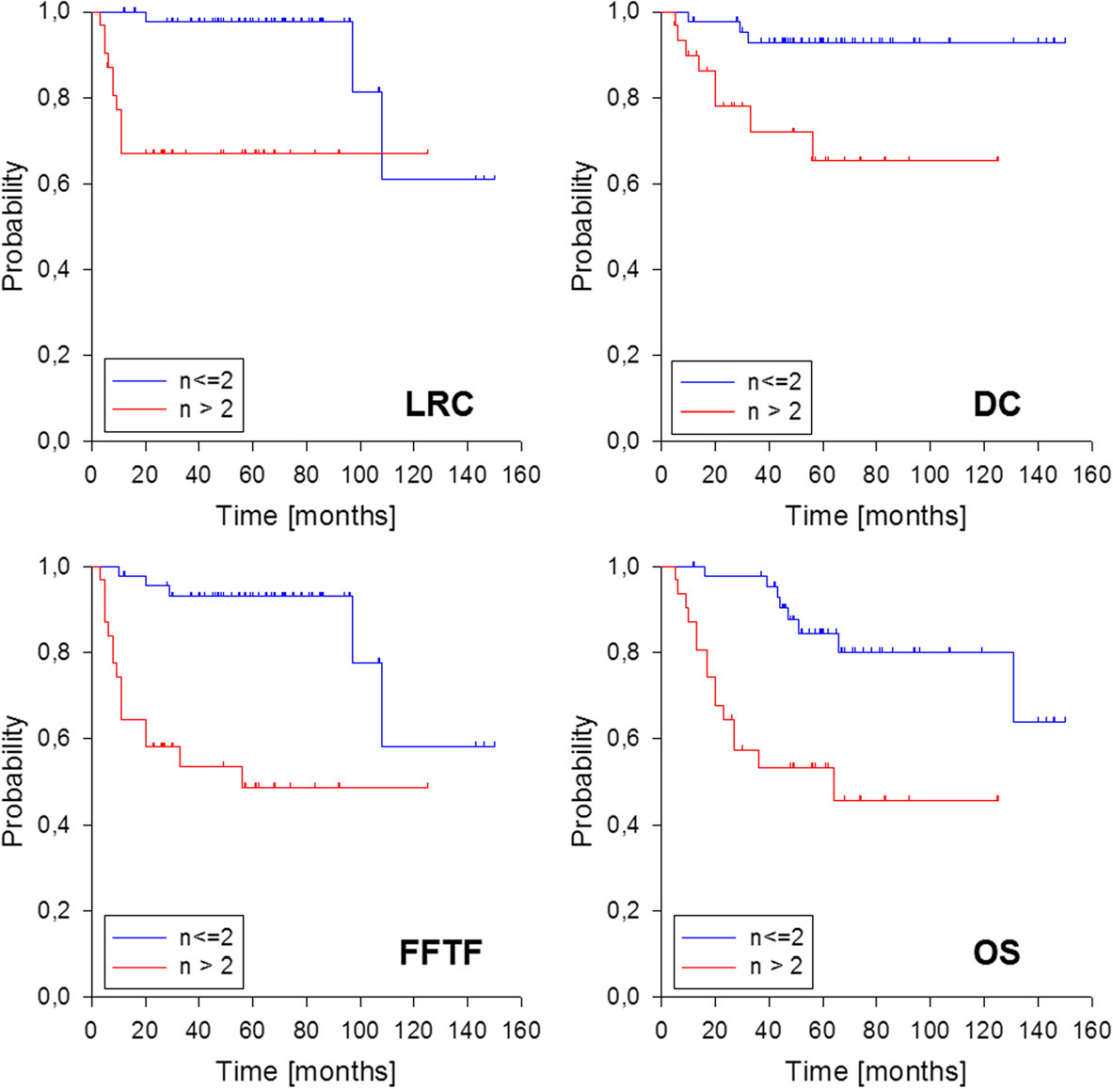
Outcome according to number of positive lymph nodes (n ≤ 2 vs >2). LRC: locoregional control, DC: distant control, FFTF: freedom from treatment failure, OS: overall survival

We observed a total of 13 locoregional failures (4 isolated local, 4 isolated neck and 5 combined), transferring into estimated 3-and 5-year locoregional control rates of 85 % (Fig. 2). The corresponding 3-and 5-year figures for local control and neck control were 90 % and 89 %. The number of positive lymph nodes (*n* > 2) and the presence of ECE were significantly associated with decreased locoregional and neck control in univariate analysis (Table 2, Fig. 3), but only the number of positive nodes remained significant according to multivariate analysis. Regarding local control, also postoperative T stage and presence of perineural invasion were significantly affected with worse outcome but only T stage remained significant in multivariate analysis (data not shown).

**Table 2:** Univariate analysis of prognostic factors

	LRC		DC		FFTF		OS	
	5-yr rate	*p* value	5-yr rate	*p* value	5-yr rate	*p* value	5-yr rate	*p* value
Age								
<58 yrs	81 %	0,144	80 %	0,803	71 %	0,349	71 %	0,932
≥58 yrs	89 %		86 %		79 %		72 %	
Gender								
male	86 %	0,598	81 %	0,439	73 %	0,745	70 %	0,503
female	83 %		91 %		83 %		81 %	
Localisation								
oral cavity	78 %	0,623	80 %	0,246	72 %	0,956	77 %	0,503
oropharynx	87 %		92 %		76 %		70 %	
Grading								
G1	100 %	0,781	100 %	0,48	100 %	0,485	100 %	0,21
G2	84 %		86 %		76 %		77 %	
G3	87 %		77 %		72 %		62 %	
pT stage								
pT1/2	88 %	0,169	87 %	**0**,**035**	78 %	0,155	75 %	0,374
pT3/4	74 %		66 %		63 %		59 %	
pN stage								
pN0/1	96 %	0,167	96 %	**0**,**05**	96 %	**0**,**017**	88 %	**0**,**006**
pN2	80 %		76 %		65 %		63 %	
clinical stage (UICC6th)								
stage 1/2/3	96 %	0,194	95 %	0,06	96 %	**0**,**023**	88 %	**0**,**009**
stage 4a	81 %		76 %		64 %		64 %	
Neck dissection								
ipsilateral	91 %	0,318	90 %	0,13	81 %	0,311	85 %	0,082
bilateral	80 %		76 %		69 %		59 %	
Number of pos. nodes								
≤2	98 %	**0**,**001**	93 %	**0**,**005**	93 %	**0**,**001**	84 %	**0**,**001**
>2	67 %		65 %		49 %		53 %	
Perineural invasion								
no	87 %	0,092	83 %	0,452	77 %	**0**,**016**	74 %	**0**,**044**
yes	60 %		80 %		40 %		30 %	
Resection margin								
R0	81 %	0,725	86 %	0,609	75 %	0,745	67 %	0,499
R1	93 %		78 %		75 %		80 %	
ECE								
no	94 %	**0**,**002**	85 %	0,309	85 %	**0**,**005**	79 %	**0**,**003**
yes	64 %		73 %		52 %		53 %	
CHT								
yes	83 %	0,373	79 %	0,335	70 %	0,179	69 %	0,134
no	90 %		90 %		86 %		80 %	
Lymph node >3 cm								
yes	94 %	0,176	89 %	0,566	84 %	0,319	77 %	0,751
no	82 %		80 %		72 %		69 %	

*LRC* locoregional control, *DC* distant control, *FFTF* freedom from treatment failure, *OS* overall survival, *yr*: year, *yrs* years, *UICC*6th union international contre le cancer staging manual 6th edition. *pos*. positive, *ECE* extracapsular extension, *CHT*: chemotherapy, *cm* centimetre, %:percentage, **bold:** significant p-values

### Distant control, FFTF and OS

Distant metastases were observed in 11 patients mainly to the lung (*n* = 8), transferring into estimated 3-and 5-year distant control rates of 85 % (Fig. 2). Higher postoperative T stage (pT3/4), higher N stage (pN2) and number of positive nodes (*n* > 2) significantly affected distant control in univariate analysis (Table 2, Fig. 3). Overall treatment failure occurred in 20 patients (9 locoregional only, 7 distant only, 4 combined), transferring into estimated 3-and 5-year FFTF rates of 77 % and 75 %, respectively (Fig. 2). The estimated 3-and 5-year overall survival rates were 80 % and 72 %, respectively (Fig. 2). Worse FFTF and OS were significantly associated with postoperative N2 stage, clinical stage 4, number of positive nodes (*n* > 2), presence of ECE and perineural invasion (Table 2, Fig. 3). According to multivariate analysis, only the number of positive nodes was significantly associated with worse FFTF while none of the factors remained significant regarding overall survival.

### Toxicity

Postoperative complications were documented in 23 patients (31 %), mainly as persistent dysphagia or aspiration requiring percutaneous endoscopic gastrostomy tubes (PEG, *n* = 13, 17 %), see Table 3. Surgical revisions were needed in 5 patients (7 %). Maximum acute toxicity grade 3 was scored in 48 patients (64 %) mainly as leukopenia or mucositis/dysphagia and grade 4 in one patient (leucopenia), see Table 4. This includes 25 patients scored as grade 3 dysphagia due to presence of a PEG at the initiation of radiation therapy (13 with PEGs placed due persistent dysphagia or aspiration postoperatively, 12 with PEGs placed prophylactically at the discretion of the treating physician), resulting in a overall acute grade 3 dysphagia rate of 52 %. If only the 50 patients without PEG at the initiation of radiation therapy were regarded, the rate of grade 3 dysphagia would have dropped to 28 %, see Table 5. Maximum late toxicity grade 3 was documented in 17 patients (23 %), mainly as long term PEG dependency (*n* = 11, 15 %), see Table 6. Patients with PEGs due to postoperative complications had a markedly increased risk of long-term tube dependency (5/13, 38 %) compared to patients who received PEGs for other reasons or not at all during radiation therapy (6/62, 10 %), see Table 5.

**Table 3:** postoperative complications

postoperative complications	number	percent
local		
dysphagia requiring feeding tube	13	17
bleeding	4	5
wound healing disturbance/fla*p* necrosis	3	4
horner’s syndrome	1	1
systemic		
MI	4	5
DVT	2	3
pulmonary embolism	1	1
tachyarrhythmia	1	1
hypertensive crisis	1	1
pseudomembraneous colitis	1	1
pneumonia	1	1
delirium	1	1
surgical revisions	5	7

*n* number, %: percentage, *MI* myocardial infarction, *DVT* dee*p* vein thrombosis, some patients had more than one postoperative complication

**Table 4:** Acute toxicities

acute toxicities	all grades		grade 3/4^a^	
	n	%	n	%
non-hematological				
dysphagia^b^	68	91	39	52
mucositis/stomatitis	61	81	8	11
weight loss	32	43	5	7
skin	64	85	3	4
nausea/vomitting	29	39	3	4
horseness/larynx edema	9	12	1	1
hearing loss	9	12	1	1
renal injury	7	9		
dry eye	1	1		
hand foot syndrome	1	1		
other	3	4	2	3
hematological				
leucoytopenia	39	52	8	10
infection	16	21	5	7
anemia	51	68	4	5
thrombocytopenia	18	24		

^a^: only one patient had a grade 4 toxicity (leucopenia), ^b^all patients included with PEGs regardless of its reason or use, some patients had more than one acute toxicity

**Table 5:** Acute and late dysphagia in relation to PEG placement

Dysphagia	all patients^a^		without PEG at start of RT	
	n	% (*n* = 75)	n	% (*n* = 50)
grade 0	7	9	7	14
grade 1	22	29	22	44
grade 2	7	9	7	14
grade 3	39	52	14	28
PEG	prior or during RT		long term after RT	
	n	%	n	%
postoperative^b^	13	17	5	38
prophylactic^c^	12	16	2	17
symptomatic^d^	7	9	0	0
none^e^	43	57	4^f^	9

*PEG* percutaneous endoscopic gastrostomy tube, *RT* radiation therapy, ^a^: all patients with PEG during RT regardless of reason for placement or use scored as grade 3 dysphagia, ^b^: patients who received PEG postoperatively due to aspiration or persistent dysphagia until initiation of RT, ^c^: patient who received PEG for prophylactic reasons prior to initiation of RT on discretion of the treating radiation oncologist, ^d^: patients who received PEG during RT due to dysphagia, ^e^: patients who did not receive a PEG during the whole course of RT, ^f^patients who needed PEG due to development of late dysphagia after completion of the full RT course

**Table 6:** Late toxicities

late toxicities	all grades		grade 3	
	n	%	n	%
dysphagia	28	37	11	15
hearing loss	9	12	3	4
xerostomia	38	51	2	3
hoarseness/laryngeal edema	7	9	2	3
abcess/fistula	2	3	2	3
trismus	8	11	1	1
osteonecrosis	1	1	1	1
taste alteration^a^	16	21		
lymph edema	8	11		
mucosal damage	4	5		
hypothyreosis	3	4		
dental damage	2	3		
skin damage	1	1		
esophageal stenosis	1	1		

^a^only grade 1 and 2 possible according to CTCAE3.0, some patients had more than one late toxicity

## Discussion

In our current analysis of 75 patients suffering from oral cavity or oropharyngeal squamous cell carcinoma, we show that encouraging locoregional control rates (5-year LRC 85 %) and overall survival rates (5-year OS 72 %) can be achieved with postoperative IMRT with or without simultaneously applied chemotherapy according to the presence of established risk factors with acceptable acute and limited late toxicities. Despite the general limitations in comparing different studies, our results seem to compare favourable with the findings of large prospective trials using similar approaches with conventional radiation techniques in head and neck cancer especially with regard to overall survival [3, 4]. For example, Bernier et al. [4] reported a 5-year local or regional recurrence rate of 18 % and a 5-year overall survival rate of 53 % in the chemoradiation arm of EORTC 22931 using a slightly different dose concept combined with cisplatin. Cooper et al. [3] described a 10-year local or regional recurrence rate of 22 % and an estimated 5-year overall survival of 46 % in the chemoradiation arm of RTOG 9501 which was similarly designed to EORTC 22931. Although the majority of patients in these studies suffered from oral cavity or oropharyngeal cancers (56-72 %), hypopharyngeal and laryngeal cancers were also included which might have affected the overall results. Further on, the use of IMRT in the present study instead of conventional radiation might have led to an improved overall survival due to decreased late toxicities, but given the retrospective nature and the small sample size of our study, it cannot be ruled out that these differences occurred simply by selection bias or randomly. However, our results are also in good accordance with other studies focusing on IMRT and/or oral cavity and oropharyngeal cancer using modern radiation techniques. For example, Chen et al. [20] analyzed 90 consecutive head and neck cancer patients treated with surgery, postoperative IMRT +/−chemotherapy and found a 2-year locoregional control rate of 80 % and a 2-year overall survival rate of 79 %. Collan et al. [5] reported a cohort of 102 patients with a stage distribution similar to ours treated by postoperative IMRT u*p* to a median dose of 60 Gy with 38 % of them receiving simultaneous chemotherapy. With a median follow u*p* of 55 months, they observed very high 5-year LRC and OS rates of ~90 % (estimated from printed curve) and 84 %, respectively. Wang et al. [11] analyzed 88 patients with primaries located in oral cavity or oropharynx of whom 44 received postoperative IMRT u*p* to 66 Gy (SD 2.2 Gy) mainly without chemotherapy with a slightly more favourable stage distribution. After a median follow-u*p* of 53 months, they found estimated 4-year LRC control and OS rates of 84 % and 71 %, respectively. In summary, postoperative IMRT with/without chemotherapy seems to result in encouraging LRC and OS rates in patients with oral cavity or oropharyngeal cancers based on our results and the limited available literature.

### Prognostic factors

Despite the relatively small sample size, we used univariate and multivariate analyses to investigate possible prognostic factors specifically for oral cavity and oropharyngeal cancer cases. Regarding locoregional control, we found that presence of ECE and number of positive nodes (*n* > 2) were associated with worse outcome in univariate analysis, but only the number of positive nodes remained significant according to multivariate Cox regression. Regarding FFTF and OS, worse outcome was associated with N2 stage, clinical stage 4 disease, number of positives nodes, presence of ECE and perineural invasion in univariate analysis, but again only the number of positive nodes remained significant according to multivariate analysis at least for FFTF. These findings were surprising at least to some extent as microscopic incomplete resection and presence of ECE have been reported as strongest factors influencing locoregional control and overall survival according to major prospective trials [3, 4, 6], while the number of positive nodes is a less established factor. For example, the combined analysis of EORTC 22931 and RTOG 9501 (which stratified patients according to slighty different risk factors) observed a significant benefit for adding chemotherapy to postoperative radiation only for the common factors (positive margin and ECE), while the presence of risk factors which have been used for risk assessment only in one of the trials (number of positive nodes ≥2, level 4/5 involvement, vascular embolisms, perineural disease, stage III/IV) had a weaker or no prognostic value at all [6]. However, our patients received chemotherapy based on the presence of the established risk factors close/positive margins or ECE, thus it cannot be ruled out that the addition of chemotherapy improved the results in those patients to a level comparable to patients at lower risk receiving postoperative RT alone and therefore mimicked their prognostic relevance with regard to the entire cohort. In contrast, the number of positive nodes did not trigger the use of additional chemotherapy, possibly maintaining its influence as highlighted by several reports supporting the significance of the number of positive nodes or the lymph node ratio as prognostic factors in head and neck cancer [21, 22]. For example Hua et al. [21] described a highly significant association between number of positive nodes (threshold ≤ 3 vs > 3) and lymph node ratio with the median overall survival in 81 patients suffering from hypopharyngeal cancer treated by surgery only. Wan et al. [22] evaluated 1510 patients with head and neck cancer treated by surgery alone, adjuvant radiation or adjuvant chemoradiation and found a strong and significant association between the number of positive nodes and locoregional control, disease specific survival and overall survival. In a subset analysis, they further described no significant difference in DSS or OS between patients with one or two positive nodes but significant worsening if three or more nodes were involved. Similarly, we found that the number of positive nodes was the only factor which significantly influenced all oncological endpoints in univariate analysis and the only factor which influenced LRC and FFTF in multivariate analysis. Interestingly the strongest discrimination in our cohort was found for the same threshold (≤2 vs > 2) as in the study by Wan et al. [22]. We performed a separate analysis using the threshold introduced by Cooper et al. (<2 vs ≥ 2) [3], which provided similar results in univariate and multivariate analysis with regard to significance but with weaker discrimination (data not shown). Given the different thresholds reported in the mentioned studies with similar results, it seems at least reasonable to assume, that locoregional control and overall survival will worsen with an increasing number of positive nodes irrespective of arbitrarily set distinctions.

### Toxicity

We observed maximum acute grade 3 toxicities in about two thirds of our patients, mainly hematological and mucositis/dysphagia. These findings are in line with the reports of other studies investigating postoperative radio (chemo) therapy in head and neck cancer. For example Cooper et al. [3] found maximum acute grade 3/4 toxicities in 34 % of the patients treated by postoperative radiation alone and 77 % if chemoradiation was used. Geretschläger et al. [23] described 66 % acute grade 3 side effects in their cohort of patients with oral cavity cancer treated by postoperative IMRT. Regarding only hematological side effects, 30 % grade 3 and 8 % grade 4 hematological toxicities were found in the chemoradiation arm of RTOG 9501 [3] and 16 % severe leucopenia was reported in the chemoradiation arm of the EORTC 22931 study [4], indicating that those toxicities are mainly driven by the (similar) chemotherapy component and not influenced by radiation technique or disease site relevantly.

The combined rate of severe acute mucositis/dysphagia was also similar with roughly 60 % in our study compared to 66 % in the chemoradiation arm of RTOG 9501 [3] and 51 % in the EORTC 22931 study [4], however we found a different distribution. While in those trials more patients had severe mucositis than dysphagia, we observed an opposite ratio. This might be due to the fact that our analysis was limited to oral cavity and oropharyngeal cancer resulting in the inclusion of swallowing structures into the high dose boost areas in most patients, however it seems also linked to the definition and grading of dysphagia. In our analysis, all patients who had a feeding tube at any time during radiotherapy were scored at least as grade 3 dysphagia, although many of them received their PEGs already due to postoperative complications or prophylactically prior to RT. If only those patients were considered without PEGs at the beginning of RT, the severe dysphagia rate would have dropped to 28 %, similarly to the 25 % in the chemoradiation arm of RTOG 9501 [3]. Interestingly, we found a clearly increased risk of long-term PEG dependency in patients who had PEG placement due to postoperative complications compared to placement for other reasons or not at all. The use of feeding tubes for nutritional support is a common but heavily discussed issue in head and neck radiation therapy. It has been estimated that 50-70 % of patients require a feeding tube during definitive chemoradiation, 15-40 % with definitive RT and 20-40 % with surgery followed by adjuvant RT [24]. Many investigators favor the prophylactic use of feeding tubes because of numerous reports describing less weight loss, improved 6-month quality of life, less morbidity and fewer hospitalizations including one randomized trial supporting this approach [25–27]. However, an increasing number of reports described high percentages of unnecessary (unused) prophylactic feeding tube placements of u*p* to 50 % [28], a higher likelihood of prolonged or permanent dependency [29] and an increased rate of esophageal strictures [30], thus favoring a more reactive approach. Unfortunately most reports focused on patients with definitive radio (chemo) therapy resulting in very limited data for patients treated with postoperative radiation [31]. Collan et al. [5] described 5 % patients with long term PEG dependency but did not report details about reasons for placement. Bastos de Souza et al. [9] found 8 % long term dependency in a cohort of 256 patients treated by surgery alone or surgery plus radiotherapy. We did not observe a major difference in prolonged PEG dependency between patients with prophylactic (17 %), symptomatic (0 %) or no placement (9 %) during radiotherapy and therefore are not able to add evidence to this issue, however we found that patients with postoperative swallowing complications requiring tube feeding are at increased risk (39 %) for prolonged PEG-dependency after postoperative radio (chemo) therapy and should be counseled accordingly.

Consistent with prior results published by our grou*p* [14, 32, 33] and several other studies [12, 13] who found decreased rates of severe xerostomia with IMRT in head and neck cancer in general or in distinct subgroups, we observed a low rate (3 %) of severe xerostomia with postoperative IMRT also in the treatment of oral cavity and oropharyngeal cancers. For example Collan et al. [5], who focused similarly on the postoperative treatment of oral cavity and oropharyngeal cancers even observed no grade 3 xerostomia with IMRT at all. Wang et al. [11] compared IMRT with conventional RT in a similar subgrou*p* and described a significant reduction of severe xerostomia in favour of IMRT (0 vs 35 %). Two randomized prospective trials have recently highlighted the value of IMRT [12, 13] by showing a significant reduction of late xerostomia compared to conventional or 3D-conformal RT in head and neck cancer in general, confirming a plethora of clinical and dosimetric studies with similar findings.

In contrast to other reports focusing on postoperative IMRT in oropharyngeal/oral cavity cancer most of our patients were treated with a simultaneously integrated boost (SIB) concept using slightly increased single doses (u*p* to 2.2 Gy) in the boost area. SIB techniques allow a slightly reduced overall treatment time and result in increased dose conformity regarding the boost area but have been associated with concerns regarding additional toxicity. Although inter-study comparisons are generally difficult and possibly flawed with several biases including patient selection or the use of different scoring systems, we did not observe markedly increased rates of acute or late toxicities compared to reports on sequential IMRT boost techniques [5, 23]. This is especially true for damage to mucosal or swallowing structures, which are regarded as possibly associated with increased single doses. However, our acute grade 3 toxicity rates of 11 % for mucositis and 28 % for dysphagia (in patients without prophylactic PEG placement) were at least comparable to Collan et al. (mucositis grade 3: 25 %) [5] and Geretschläger et al. (mucositis grade 3: 36 %, dysphagia grade 3: 34 %) [23] using strictly sequential boosts. Regarding late dysphagia, our rate of 15 % was slightly increased compared to 5-9 % in the mentioned reports [5, 23], however Collan et al. [5] treated their patients with a considerably lower median total dose compared to our study and Geretschläger et al. [23] included only patients with oral cavity tumors probably resulting in lower doses to the swallowing structures. Gupta et al. [12] recently used an integrated boost concept very similar to ours in a prospective phase randomized trial and reported very low rates of grade 3 mucositis (6 %) and dysphagia (10 %). Finally, Spiotto et al. compared IMRT using sequential and integrated boost concepts in advanced head and neck cancers and reported significantly reduced acute grade 3 mucositis for the integrated boost grou*p* [34]. In summary, the use of an integrated boost technique with slightly increased single doses does not seem to result in markedly increased toxicities compared to sequential boosting techniques.

Obviously our study has some limitations, mainly its retrospective nature and its limited number of patients. Nevertheless, it describes a homogenous cohort of consecutive patients with oral cavity or oropharyngeal cancer treated uniformly with postoperative IMRT with or without chemotherapy. Thus the data may add valuable information to the limited body of evidence specifically addressing this subgrou*p* of patients.

## Conclusion

In summary, postoperative IMRT with or without chemotherapy achieves excellent locoregional control and good overall survival with acceptable acute and low late toxicity rates. Patients who needed PEG placement due to postoperative complications were at higher risk for prolonged PEG dependency than patients with prophylactic, symptomatic or no PEG placement during radiotherapy. While the selective addition of chemotherapy may have compensated the influence of established risk factors like positive margins or extracapsular extension, the number of positive lymph nodes (*n* > 2) remained a strong prognostic factor for all endpoints including locoregional control and freedom from treatment failure.

## Abbreviations

CHT: chemotherapy
CT: computed tomography
CTCAE: common toxicity criteria adverse events
CTV: clinical target volume
DC: distant control
DSS: disease specific survival
DVH: dose volume histogram
DVT: dee*p* vein thrombosis
EBRT: external beam radiotherapy
ECE: extracapsular extension
EORTC: European organisation for research and treatment of cancer
FFTF: freedom from treatment failure
Gy: gray
IMRT: intensity-modulated radiation therapy
kV-CT: kilovoltage computed tomography
LC: local control
LRC: locoregional control
MI: myocardial infarction
MLC: multi leaf collimator
MRI: magnetic resonance imaging
NC: neck control
OS: overall survival
PEG: percutaneous endoscopic gastrostomy tube
PTV: planning target volume
RT: radiation therapy
RTOG: radiation therapy oncology group
SCC: squamous cell carcinoma
SD: single dose
SIB: simultaneously-integrated boost
TD: total dose
UICC: union international contre le cancer
